# Conflict of Interest Disclosures for Clinical Practice Guidelines in the National Guideline Clearinghouse

**DOI:** 10.1371/journal.pone.0047343

**Published:** 2012-11-07

**Authors:** Susan L. Norris, Haley K. Holmer, Lauren A. Ogden, Shelley S. Selph, Rongwei Fu

**Affiliations:** 1 Department of Medical Informatics and Clinical Epidemiology, Oregon Health & Science University, Portland, Oregon, United States of America; 2 Department of Public Health and Preventive Medicine, Oregon Health & Science University, Portland, Oregon, United States of America; 3 Department of Emergency Medicine, Oregon Health & Science University, Portland, Oregon, United States of America; Yale University School of Medicine, United States of America

## Abstract

**Background:**

Conflict of interest (COI) is an important potential source of bias in the development of clinical practice guidelines (CPGs) and high rates of COI among guideline authors have been reported in the past. Our objective was to report current rates of disclosure and specific author COI across a broad range of CPGs and to examine whether CPG characteristics were associated with the presence of disclosures and of conflicts.

**Methods and Findings:**

We selected a random sample of 250 CPGs listed in the National Guideline Clearinghouse on November 22, 2010, representing approximately a 10% sample of guidelines listed in the NGC on that date. We abstracted information on author COI from each CPG and examined predictors of the disclosures and COI using a logistic generalized estimating equation regression model. 87% of organizations developing guidelines had a CPG-specific policy, however, 40% of CPGs did not indicate that they had collected disclosures from guideline authors. In addition, 42% of organizations that did collect author disclosures did not have those disclosures available in the public domain. Of CPGs where we had disclosures for all authors, 60% had one or more authors with a conflict. On average, 28% of the authors of CPGs with available disclosures had a COI. Guidelines that were published in journals with an impact factor greater than 5.0 were more likely to have one or more authors with a COI than guidelines not published in journals.

**Conclusions:**

Rates of disclosure of author COI and the public availability of that information are unacceptably low, however rates of COI among guideline authors may have decreased in recent years. Continued efforts are needed to establish and enforce optimal COI policies in clinical practice guideline development in order to minimize the risk of bias associated with those conflicts.

## Introduction

Conflict of interest (COI) is an important potential source of bias in the development of clinical practice guidelines (CPGs). A COI is a set of conditions in which professional judgment concerning a primary interest (such as the health and wellbeing of a patient or the validity of research), may be unduly influenced by a secondary interest [1]. Physician-industry relationships [2] and industry funding of research [3], [4] are frequent and increasing in prevalence. There are data suggesting an association between author or funder COI and study outcomes [5], [6], [7], [8], [9], between industry relationships and physician behavior [10] or expressed opinions [11], [12], and between COI and conclusions in systematic reviews [13].

CPGs are “statements that include recommendations intended to optimize patient care that are informed by a systematic review of evidence and an assessment of the benefits and harms of alternative care options” [14]. CPGs can influence the care delivered by a large number of healthcare providers and thus potentially the outcomes of patients [15]. The quality of CPGs is therefore critically.

important: high-quality, or trustworthy guidelines promote the use of effective clinical services, decrease undesirable practice variation, reduce the use of services that are of minimal or questionable value, increase the use of effective but underused services, and target services to populations most likely to benefit [16].

Data on the prevalence of industry relationships of CPG sponsors and authors are either limited to specific clinical areas [17], [18], [19] or dated [20], [21], [22]. A study published in *Nature* in 2005 [21] reported that 49% of 215 CPGs in the National Guideline Clearinghouse provided no disclosures of COI, and that 43% of CPGs with disclosures had one or more authors who were paid by industry for speaking engagements. Of the 685 authors of these CPGs, 35% disclosed an interest in a company relevant to the topic of the guideline. More recently, Neuman and colleagues [18] reported that 48% of panel members producing CPGs on the management of diabetes or hyperlipidemia disclosed a COI while 27% of members were not given the opportunity to disclose conflicts. 36% of guidelines (n = 14) did not provide an opportunity for panelists to publicly declare financial COI [18].

The Institutes of Medicine (IOM) report on Conflict of Interest in Medical Research, Education, and Practice [23] noted that additional information is needed on the prevalence of COI in CPGs, and on the potential effects of such conflicts. The objective of this study was to address this knowledge gap by reporting on current practices of COI disclosure and on author COI across a broad range of CPGs. In addition, we examined whether characteristics of guideline topics, sponsors, developers, and publications were associated with the presence of disclosures and of reported conflicts.

## Methods

We selected a random sample of 250 CPGs listed in the National Guideline Clearinghouse (NGC) (http://www.guideline.gov) on November 22, 2010, representing approximately a 10% sample of guidelines listed in the NGC on that date. The NGC is funded by the Agency for Healthcare Research and Quality (AHRQ), and was established in January, 1999 “to provide physicians and other health professionals, health care providers, health plans, integrated delivery systems, purchasers, and others an accessible mechanism for obtaining objective, detailed information on clinical practice guidelines and to further their dissemination, implementation, and use” [24]. The inclusion criteria for guidelines within the NGC are: 1) The clinical practice guideline contains systematically developed statements that include recommendations, strategies, or information that assists physicians and/or other health care practitioners and patients to make decisions about appropriate health care for specific clinical circumstances. 2) The clinical practice guideline was produced under the auspices of medical specialty associations; relevant professional societies, public or private organizations, government agencies at the Federal, State, or local level; or health care organizations or plans. 3) Corroborating documentation can be produced and verified that a systematic literature search and review of existing scientific evidence published in peer reviewed journals was performed during the guideline development. 4) The full text guideline is available upon request in the English language. 5) The guideline was developed, reviewed, or revised within the last 5 years.

Random numbers for the selection of our cohort of CPGs were obtained from Random.org (http://www.random.org). Because CPGs are continuously added and archived in the NGC, CPGs identified in the original cohort were not always available at the time of data abstraction. In that situation, we selected the CPG corresponding to the next random number in our sequence.

One author abstracted information from each CPG and from the corresponding summary in NGC, into a pre-specified template in Microsoft Excel®, and those data were checked by a second author. We examined the NGC summaries because in some circumstances they provided more detailed information on CPG author COI, (upon submission of a guideline to the NGC, guideline developers are asked to disclose COI if none was provided in the submitted CPG). We also looked for disclosure information in CPG supplemental documents if any were referenced.

Data were abstracted on: 1) where the CPG was published; 2) the presence of a COI policy for the developer of the CPG and the journal where the CPG was published (if applicable); 3) the number of authors or panel members responsible for the recommendations in the CPG; 4) specific disclosures for each author of each CPG. We stratified disclosures as financial and nonfinancial, and further categorized the financial conflicts into one or more categories (advising, consulting, research, patents/royalties, stock/equity, gifts, other) based in part on the frameworks proposed by Papinakalou [22] and Cosgrove [25]. Nonfinancial COI included any interests that are not usually assessed in terms of their monetary value, including intellectual, academic, and professional interests.

If conflicts were listed for some but not all of the CPG authors, and there was no mention of all authors having disclosed, we assumed that all authors had provided COI statements, and that those authors without disclosures had no conflicts. If the CPG indicated that authors and/or panel members with COI were excluded from participating in the CPG development process, we assumed that none of the authors had COI.

This study was exploratory and descriptive, thus our approach to data analysis and synthesis was largely qualitative. We examined four outcome variables: 1) the presence of any disclosure in the CPG (including disclosures of no COI); 2) whether the presence of COI could be assessed for the CPG (Yes versus No, the latter including CPGs with no disclosures and CPGs with disclosures that were not publically available); 3) whether one or more CPG authors had any disclosed COI; and 4) the percentage of CPG authors with any disclosed COI. For outcomes 1) and 2), the analysis used the entire CPG sample (n  = 250). Outcomes 3) and 4) involved the subset of CPGs where the specific conflicts were disclosed and available to us.

The association of pre-specified predictor variables with the four outcomes above was examined. The predictor variables included year of publication or guideline update; funder; type of organizations that developed the CPG and type of organizations that funded the CPG (both classified as government, academic, professional organization, and combined); number of organizations that developed the CPG (1, 2, or 3); number of organizations that funded the CPG (1 or more than 1); whether the funder was the same as the developer of the CPG; types of interventions contained in the CPG (any recommendation regarding drug therapy versus no drug-related recommendations); whether the CPG was published in a journal, and if it was, whether the journal had a CPG-specific COI policy; the 2010 impact factor of the journal) [26]; and the country where the CPG originated. Given the exploratory nature of this study, only significant variables were included in the final model.

Characteristics of the CPGs and the organizations producing them were summarized using descriptive statistics. To assess the association between outcomes 1) through 3) and the pre-specified independent variables, we used a logistic generalized estimating equation (GEE) regression model. For outcome 4), the percentage of CPG authors with a COI, we used a linear GEE model. Since some organizations within our cohort developed multiple CPGs, the GEE approach was used to account for correlations among multiple CPGs.

## Results

### Characteristics of Organizations Producing CPGs

The 250 CPGs in our cohort were developed by 97 different organizations worldwide, with over half (59.6%) originating in the US (Table 1). On the date or our search, NGC listed between 1 and 17 guidelines (median 2) from 97 different organizations. 188 of the CPGs were developed by a single organization, while 44 were co-developed by two organizations and eight by three. 142 of the CPGs in our cohort were the original release of the guideline: the remainder were updates. The majority of CPGs (62%) were published in a journal; the others were available only online or in hard-copy format. Professional organizations were the most frequent developer of the guidelines in our cohort (69%). Authors were individually listed in 233 CPGs (mean number of authors 15, range 1–92); the remaining 17 CPGs were authored by guideline development groups.

**Table 1:** Characteristics of the included clinical practice guidelines (n = 250).

Guideline characteristic	Results
Year released or updated [Number (%)]	2005: 3 (1.2)
	2006: 75 (30.0)
	2007: 38 (15.2)
	2008: 74 (29.6)
	2009: 51 (20.4)
	2010: 9 (3.6)*
Number of authors (n = 233)	Range: 1–92; Median: 12; Mean: 15; In 17 guidelines there was no list of authors
Country [Number (%)]	US: 149 (59.6)
	Canada: 38 (15.2)
	Western Europe: 47 (18.8)
	International: 4 (1.6)
	Other: 12 (4.8)
Publication site [Number (%)]	Journal: 155 (62.0)
	Organization web-site or hard-copy publication: 95 (38.0)
Journal impact factor (n = 112 unique journals) [Number (%)]	<5 or no impact factor: 55 (49.1)
	≥5 and <10: 31 (27.7)
	≥10: 26 (23.2)
Developer [Number (%)]	Government: 59 (23.6)
	Professional organization: 173 (69.2)
	Academic institution: 14 (5.6)
	More than one type of organization: 4 (1.6)
Relationship between the guideline developerand the funder [Number (%)]	Same: 205 (82.0)
	Different: 40 (16.0)
	Unknown: 5 (2.0)

n = 250 guidelines unless otherwise indicated.

(*) Includes one guideline from 2011 because at the time of abstraction, one guideline that was initially identified had been removed from the National Guideline Clearinghouse and thus the next guideline in our sequence of random numbers was selected, which was dated 2011.

### Disclosure Statements and Conflicts of Interest among CPGs and CPG Authors

**Figure 1:**
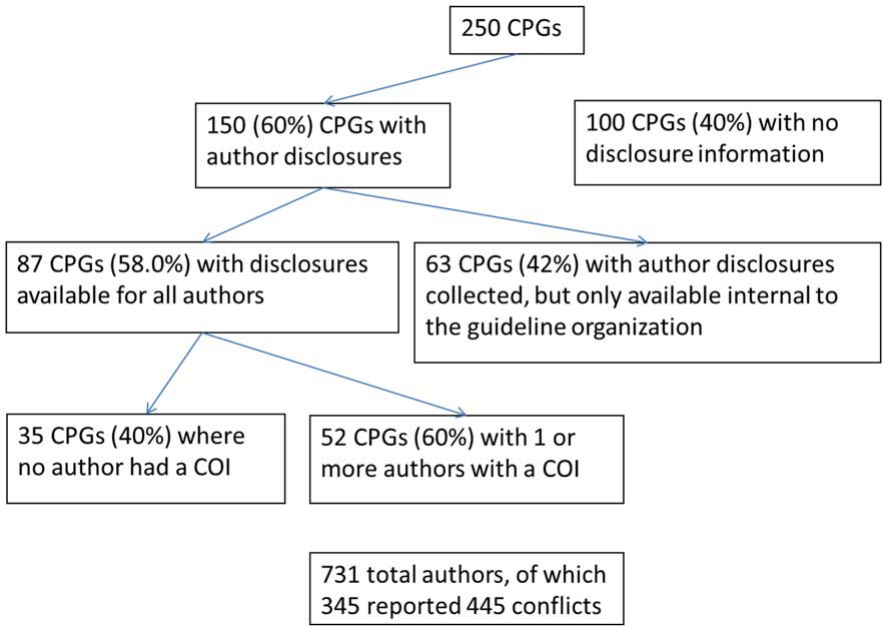
Study flow diagram. Abbreviations: COI, conflict of interest; CPGs, clinical practice guidelines.

In our cohort, 217 (86.8%) of the guidelines were developed by organizations with COI policies specific to the development of CPGs. The majority of the CPGs (60.0%, n = 150) reported disclosure statements either in the CPG itself or in the NGC summary (Figure 1). For 63 of these 150 guidelines, however, these statements were not publically available. Thus, in total, only 87 (34.8%) of the 250 CPGs provided publicly available COI information on all authors.

Of the 87 CPG’s with public disclosures, 35 (40.2%) had no authors with a COI. Of those CPGs with one or more authors with a COI (n = 52), the mean proportion of authors with COI was 46.8% (standard deviation 5.4%) (range 4.2% to 100%) (Table 2).

**Table 2:** Conflicts of interest among clinical practice guidelines and their authors.

Characteristic	Result
Guideline developer has a CPG-specific COI policy) [Number (%)] (n = 250)	Yes: 217 (86.8)
	No: 33 (13.2)
Guideline has author disclosures (with or without COI) [Number (%)] (n = 250)	Yes: 150 (60.0) [Publically disclosed: 87 (34.8); Internally disclosed only: 63 (25.2)]
	No: 100 (40.0)
Proportion of authors of each guideline with COI (%) (n = 87 CPGswith public disclosures for all authors)	Mean (SD): 28.0 (32.3); Median: 11.8; Range: 0 to 100
Guideline has 1 or more authors with COI (%) (n = 87)	Yes: 52 (60.0)
	No: 35 (40.0)
Types of COI disclosed in CPGs* [Number of CPGs (%)] (n = 52 CPGs with1 or more authors with a disclosed conflict)	Advising/consulting: 49 (94.2)
	Research: 40 (76.9)
	Stocks/equity: 12 (23.1)
	Gifts: 4 (7.7)
	Patents/royalties: 3 (5.8)
	Intellectual: 3 (5.8)
	Other: 3 (5.8)
Proportion of authors of each guideline with a conflict (%) (n = 52 CPGswith 1 or more authors with a conflict)	Mean (SD): 46.8 (5.4); Median: 48.9; Range: 4.2–100
Types of COI** disclosed by 325 authors with conflicts [Number (%)](n = 445 unique conflicts)	Advising/consulting: 220 (49.4)
	Research: 165 (37.1)
	Patents/royalties: 3 (0.7)
	Stocks/equity: 20 (4.5)
	Gifts: 6 (1.3)
	Intellectual: 6 (1.3)
	Other: 25 (5.6)

(*) % add to more than 100 because CPG authors can disclose more than one type of COI.

(**) Examples of activities for the types of COI: Advising/consulting: serving on an advisory board and/or executive committee, or receiving consulting fees, honoraria, speaking fees, or providing expert testimony; Research: receiving research grants, salary support; Patents/royalties: receiving income from patents or royalties; Stocks/equity: owning stock or equity in a company; Gifts: receiving gifts, travel, samples, or educational materials; Intellectual: publishing on a topic, providing unpaid advocacy; Other: time devoted to specific procedures, spousal employment.

Abbreviations: COI, conflict of interest; CPG, clinical practice guideline, SD; standard deviation.

The most common types of conflicts among the 52 guidelines with one or more authors with a conflict, were payments for advising/consulting (94% of these guidelines) and research (76.9%) (Table 2). These 52 CPGs had a total of 731 authors, of whom 325 (44.5%) disclosed 445 unique conflicts, including advising/consulting (49.4% of conflicts) and research (37.1%). Only three CPGs mentioned non-financial/intellectual COI, and six of the 445 total disclosures (1.3%) were for intellectual COI.

### Predictors of Disclosures and Conflicts of Interest among CPGs and CPG Authors

Several variables were found to significantly predict whether a CPG provided any disclosures for authors (Table 3), including whether the guideline was published in a journal and impact factor of the journal (*P* = 0.0014). CPGs published in a journal with an impact factor less than 5 were less likely to provide disclosures, compared with CPG not published in a journal (odds ratio [OR] 0.311; 95% confidence interval [CI] 0.127, 0.759; *P* = 0.011). Whether the organization had a policy for COI (*P* = 0.015), the number of organizations that developed the CPG (P = 0.031), and the country of the organization developing the CPG (*P* = 0.021) all predicted whether the guideline provided disclosures. CPGs developed by two organizations were less likely to provide disclosures than those developed by three organizations (OR 0.111; 95% CI 0.022, 0.568; *P* = 0.0085).

**Table 3:** Association between clinical practice guideline characteristics and disclosures and conflicts (n = 250 guidelines).

	Presence of any disclosure*		Whether COI could be assessed**	
CPG Characteristic	OR (95% CI)	P-value	OR (95% CI)	P-value
**Published in a journal and IF**	NA	0.014***	NA	0.0004
Not published in a journal (reference)				
Published in a journal with IF <5	0.311 (0.127, 0.759)	0.011	1.400 (0.604, 3.244)	0.4308
Published in a journal with IF between 5 and 10	2.086 (0.588, 7.399)	0.254	5.873 (1.939, 17.790)	0.0019
Published in a journal with IF >10	2.914 (0.815, 10.422)	0.0996	7.662 (2.495, 23.534)	0.0004
Whether the organization has a COI policy(Yes vs. No)	4.932 (1.367, 17.801)	0.0150		
**Country of the organization**	NA	0.0214***		
US (reference)				
Canada	5.508 (1.103, 27.496)	0.0376		
Europe	3.570 (1.295, 9.841)	0.0141		
International	0.676 (0.221, 2.062)	0.4897		
Others	2.595 (0.374, 17.994)	0.3332		
**Number of organizations that** **developed the CPG**	NA	0.0312*		
1 (Reference)				
2	3.903 (0.996, 15.297)	0.0507		
3	0.433 (0.141, 1.328)	0.1423		
**Number of organizations that funded** **the CPG** (More than one vs. one)			4.141 (1.538, 11.152)	0.0051

Abbreviations: CI, confidence interval; COI, conflict of interest; CPG, clinical practice guideline; IF, impact factor; NA, not applicable; OR, odds ratio.

(*) The presence of any disclosure in the clinical practice guideline (including disclosures of no COI, Yes versus No).

(**) Whether the presence of COI could be assessed for the CPG (Yes versus No, the latter including CPGs with no disclosures and CPGs with disclosures that were not publically available).

(***) P-value for the variable overall.

CPGs developed by a Canadian (*P* = 0.038) or European organization (*P* = 0.014) were more likely to provide disclosures than those developed by a US organization.

In the final model examining whether the presence of COI could be assessed in a CPG, two variables were significant: whether the CPG was published in a journal and the journal impact factor (*P* = 0.0004), and the number of organizations that funded the CPG (*P* = 0.0051) (Table 3). Compared to CPGs not published in a journal, CPGs published in a journal with an impact factor greater than 10 were more likely to provide disclosures that were accessible to the reader (OR 7.662; 95% CI 2.495, 23.534; *P* = 0.0004). CPGs funded by more than one organizations were more likely to provide disclosures than those funded only by one organization (OR 4.141; 95% CI 1.538, 11.152). Only three CPGs were funded by three organizations, hence for this analysis these were combined with CPGs funded by two organizations.

For the analysis of the subset of CPGs where we could determine the presence or absence of author COI (n  = 87), only whether the guideline was published in a journal and the impact factor were significantly associated with the presence of any COI in the final model (*P* = 0.0001). The number of developers, number of funders, and the type of funder and developer were not significant in the final model (P>0.05). Compared to authors of CPGs not published in a journal, one or more authors of CPGs published in a journal with an impact factor greater than five were more likely to have a COI (OR 48.794; 95% CI 8.678, 274.366; *P*<0.0001), while authors of CPGs published in a journal with an impact factor less than 5 showed no significant difference (OR 2.911; 95% CI 0.570, 14.862; *P* = 0.20). Due to the small sample size, CPGs published in a journal with an impact factor between 5 and 10 and greater than 10 were combined into one group in this analysis.

Similarly, whether the CPG was published in a journal and impact factor of the journal were also significant in the final model for the percentage of authors with COI (n = 87, *P* = 0.0004). The percentage of authors who reported COIs for CPGs published in a journal with an impact factor greater than 5 was 33.3% higher than the percentage of authors of CPGs not published in a journal (95% CI 17.2%, 49.4%; *P*<0.0001). There was no significant difference in percentage of authors with COIs between CPGs published in a journal with an impact factor less than five and CPGs not published in a journal (mean difference 11.3%; 95% CI 14.4%, 37.0%; *P* = 0.384).

## Discussion

It appears that some progress has been made by CPGs developers to improve transparency through disclosures by CPG authors over the last two decades. Choudhry and colleagues [20] reported that only 2 of 44 CPGs published between 1991 and 1999 included author disclosures. Similarly, Papanikolaou and coauthors [22] reported that only seven of 40 guidelines published in six major clinical journals in 1999 provided disclosures. Tregear [27] reported that the proportion of summaries in NGC with financial relationships reported increased from about 20% in 1999 to 50% in 2006, although these summaries contain information supplied by guideline developers in response to a specific request for COI information, and thus may be more complete than information contained only within the guideline document. More recently, Neuman and colleagues [18] reported that 64% of 14 CPGs on diabetes or hyperlipidemia provided public author disclosures and 17 recent CPGs by the American college of Cardiology and the American Heart Association all provided disclosures [19]. The current study also suggests that disclosure rates, although still suboptimal, have improved.

This apparent improvement in disclosure rates may be attributed in part to increased awareness of the potential importance of COI in primary medical research and in derivative products such as systematic reviews and clinical practice guidelines [14], [23], [28]. There have been major efforts in the last 5 years to devise and update policies on COI disclosure and management by journals, academic institutions, government agencies, and professional organizations [23], [29], [30]. In 2011 the IOM released a report with standards for guideline development based on an expert consensus process and evidence when available [14]. One of the eight standards focuses on the management of COI and a second standard relates to the composition of the guideline development group. The IOM recommends disclosure of all potential and confirmed members of guideline panels, divestment of financial interests that could be affected by the guideline, exclusion (when possible) of individuals with COI, and a multidisciplinary and balanced guideline panel. Guideline organizations are beginning to incorporate these standards into their processes and policies [31] and thus disclosure rates and guideline author COI may continue to improve.

Disclosures of COI by CPG panel members should be readily available to all users of a guideline in order to assess the risk of bias and the credibility of the guideline. It is therefore concerning that 42% of guidelines with disclosures did not make those disclosures available in the public domain. It is also not sufficient in our view to have disclosures only in the NGC summary and not in the CPG itself, as many users will not access the summary.

We report a lower proportion of conflicted CPG authors than have prior studies [17]–[21], [25], [32]–[34]. In two recent studies examining small cohorts of CPGs [18], [19], the percentage of authors with COI was 56% [19] and 65% [18] (among authors with publically available disclosures). Older studies report even higher rates of COI among guideline panel authors [17], [25], [32], [33].

It is difficult to compare rates of COI for panel members across guidelines, however. Studies examining rates of COI in CPGs generally involve small, select cohorts with variations in publication sites (peer reviewed journal or web-based), policies on public availability of disclosures, disclosure forms and instructions (particularly relevance of the disclosure to the content of the CPG), and management of disclosed conflicts [35].

Although rates of disclosure of COI may be improving, a significant proportion of CPGs are developed by panels with one or more authors with a COI. There are likely a number of factors contributing to these continued high rates. Industry accounts for more than half of biomedical research funding and is continuing to increase in proportion to other funding sources [36], so clinical experts who conduct research and who participate in guideline development may be receiving significant funding from industry. Guideline authors who are asked to complete disclosure forms may be providing more complete and accurate disclosures in view of an increased awareness of COI, anticipation of scrutiny by journal editors and readers, and the use of the International Committee of medical Journal Editors (ICMJE) standardized disclosure form [29]. Guidelines on specialized medical treatments may recruit from a small number of individuals with the relevant expertise, such that involving unconflicted individuals may not be possible.

Several factors were noted to be associated with whether or not CPGs had author disclosures. Journals with impact factors less than five had disclosures less commonly than CPGs that were not published in journals, suggesting that these journals either did not have COI polices or did not adhere to existing policies. Guidelines produced by Canadian organizations were more likely to have disclosures than US organizations, suggesting room for improvement among US-based organizations. Disclosures do appear to be occurring more frequently in later years of our 5-year examination, suggesting that positive changes are occurring.

CPGs published in journals with an impact factor greater than five were associated with an increased proportion of authors with COI. The reasons for this are unclear, but in addition to actually having more conflicts, authors might be more consistent in reporting COI in these journals due to the use of ICMJE forms, author experience, journal editor attention and pressure, and policy-specific influences such as a requirement to update COI disclosures periodically.

This study focuses on disclosures of COI by CPGs authors, however, the central issue for guideline quality is the risk of bias and the diminished credibility related to the secondary interests held by guideline developers. The relationship between disclosures and biased recommendations in CPGs is complex and poorly understood. The effect of COI on conclusions in CPGs is unknown [37] as also is the effect of disclosing COI on the authors and the users of CPGs. We did not examine the management of disclosures for each of the CPGs in our cohort. It is possible that some of the effects of the disclosed conflicts were mitigated with procedures and approaches carried out by each organization during CPG development.

There are limitations to our approach. Although we examined COI statements from both the CPG itself and the statements provided to the NGC, disclosures in publications may not always be accurate [38], [39], [40], and the information in NGC may have limitations reflecting the quality of source documents used to determine COI [23]. Additionally, we did not request author disclosures from organizations that did not provide these disclosures in the public domain as these were usually listed as available to members of that organization only. We examined all authors or panel members for each guideline, irrespective of their role on each panel; it is possible that the chair or co-chair of each guideline group might have played a dominant role in deliberations, and thus their COI might be more important than for other panel members. We did not examine the sources of funding for development of each CPG, so it is possible that funders may also have had a role in formulating recommendations in CPGs.

Our findings may be generalizable to other guidelines within the NGC, given our random, 10% sample. Applicability of our findings to CPGs not contained within NGC may be limited, however. For example, guidelines not published in English or those without an underlying systematic review may have different rates of disclosure and COI than those in our cohort.

Although financial interests are usually the most obvious, intellectual interests are increasingly recognized and may be powerful motivators for researchers, systematic reviewers, and guideline authors [41], [42]. Intellectual COI has been defined as “academic activities that create the potential for an attachment to a specific point of view that could unduly affect an individual’s judgment about a specific recommendation” [43]. Intellectual interests include benefits the advancement of medical science, career advancement, fulfillment of a desire to do good, opportunity to publish, notoriety, future success in obtaining grant funding for research, and increased sense of self-worth [44]. Very few CPGs in our cohort disclosed nonfinancial COI and future efforts in COI transparency need to address this.

It is clear that much improvement is still needed in the rates of disclosure of author COI, in the public availability of that information, and in the unacceptably high rates of COI among guideline authors. CPGs can be an important tool for improving patient care, and as such, continued efforts are needed to optimize their quality by increased transparency and by minimizing potential sources of bias.
