# Correction: Conflict of Interest Disclosures for Clinical Practice Guidelines in the National Guideline Clearinghouse

**DOI:** 10.1371/annotation/1b125769-1f74-4225-94b8-bd2c80adae1f

**Published:** 2013-08-12

**Authors:** Susan L. Norris, Haley K. Holmer, Lauren A. Ogden, Shelley S. Selph, Rongwei Fu

Figure 1 has some arrows that are missing from the image. Please see the correct version of the figure at the following link:

http://plosone.org/corrections/pone.0047343.g001.cn.tif

